# Comparison of RPR and ELISA with TPHA for the Diagnosis of Syphilis: Implication for Updating Syphilis Point-of-Care Tests in Ethiopia

**DOI:** 10.1155/2018/2978419

**Published:** 2018-07-08

**Authors:** Markos Negash, Tadelo Wondmagegn, Demeke Geremew

**Affiliations:** Department of Immunology and Molecular Biology, School of Biomedical and Laboratory Sciences, University of Gondar, Gondar, Ethiopia

## Abstract

**Background:**

Syphilis is a sexually transmitted disease (STD) caused by the spirochete *Treponema pallidum*, and it persists to be a major public health problem in Africa, including Ethiopia. Syphilis diagnosis is made by either nontreponemal or treponemal approaches, though in developing countries the diagnosis relies mostly on nonspecific tests due to several reasons. Thus, the objective of this study was to assess the sensitivity, specificity, predictive values, and agreement of rapid plasma reagin (RPR) and enzyme-linked immunosorbent assay (ELISA) with *Treponema pallidum* hemagglutination assay (TPHA) as a gold standard for the diagnosis of syphilis.

**Results:**

The sensitivity, specificity, and positive and negative predictive values of ECOTEST-RPR were 100%, 80.8%, 76.2%, and 100%, respectively. However, the sensitivity, specificity, and positive and negative predictive values of DIALAB-ELISA were 98.4%, 94.9%, 92.3%, and 98.9%, respectively. The agreement between DIALAB-ELISA and Randox-TPHA was excellent (kappa value: 0.96) as compared to ECOTEST-RPR and Randox-TPHA assay (kappa value: 0.88).

**Conclusion:**

We found a characteristically variable performance of DIALAB-ELISA test and the currently available traditional ECOTEST-RPR test in the study area. The use of ECOTEST-RPR as a diagnostic test is confronted by its false positivity. Thus, neither the ECOTEST-RPR nor the DIALAB-ELISA test stands on its own to be used either as screening or confirmatory test for syphilis diagnosis. Consequently, thorough studies should be conducted aiming on a change of the current diagnostic scheme in the community.

## 1. Background

Syphilis is a sexually transmitted disease (STD) caused by the spirochete *Treponema pallidum* (*T. pallidum*) which can be spread by sexual contact, by blood transfusion, and via vertical transmission [1]. Syphilis affects 12 million people annually and results in significant morbidity if not mortality. In sub-Saharan Africa, syphilis remains a serious public health problem. Prevalence of active syphilis infection among African countries showed 12.8% in Tanzania and Kenya [2, 3]. But the magnitude of syphilis among blood donors in Gondar (Ethiopia) was 1.3% in 2010 [4].

Syphilis diagnosis can be made by several approaches. In addition to the mainstay serological diagnostic method, dark-field microscopy by which the spirochete is examined and observed from the lesion under dark-field microscope is also another method [5–7]. *T. pallidum*, the etiological agent of syphilis, produces at least two types of antibodies in human infections: treponemal antibodies that can be detected by fluorescent treponemal antibody absorption (FTA-ABS) and nontreponemal antibody (reagin) that can be detected by RPR antigen card or VDRL test. Nontreponemal tests such as the venereal disease research laboratory (VDRL) and rapid plasma reagin (RPR) are based on the reaction of cardiolipin with nonspecific antibodies produced in response to syphilitic infection [8]. However, this test lacks sensitivity and specificity due to several reasons including pregnancy, autoimmune disorders, infections, and stages of syphilis infection [9, 10]. Therefore, treponemal-specific tests like enzyme immunoassay (EIA), *T. pallidum* hemagglutination assay (TPHA), microhemagglutination, fluorescent treponemal antibody absorption test (FTA-abs), and the enzyme-linked immunosorbent assay (ELISA) that detect IgG antibodies to antigenic components of *T. pallidum* are used primarily to confirm the diagnosis of syphilis in patients with a reactive nontreponemal test [11, 12]. Despite the availability of relatively sensitive tests and affordable treatment, the disease remains a global health problem [13, 14].

Syphilis remains a major cause of reproductive morbidity and poor pregnancy outcomes in developing countries including Ethiopia. In 80% of infected pregnant women, it results in stillbirth and spontaneous abortion (40%), perinatal death (20%), and serious neonatal infections and low-birth weight babies (20%) [15–17]. Syphilis has also acquired a new potential for morbidity and mortality through an association with increased risk for HIV infection [18].

Screening of pregnant women, blood donors, and social workers (drivers and newly employed social workers) for syphilis is a routine activity at all healthcare institutions in Ethiopia. For this purpose, due to cost effectiveness, RPR which has questionable test performance is usually used as a screening tool in Ethiopia. However, despite reports of diagnostic performance provided by the manufacturers, data on test performance of RPR in the study area remain limited. Thus, the objective of this study was to assess the sensitivity, specificity, predictive values, and agreement of ECOTEST-RPR and DIALAB-ELISA with Randox-TPHA assay as a gold standard for syphilis diagnosis among syphilis-suspected patients attending the University of Gondar Hospital (UGH), Northwest Ethiopia.

## 2. Methods

### 2.1. Study Design, Period, and Area

Facility-based cross-sectional study was conducted in UGH, from November 2015 to June 2016. The University of Gondar Hospital is one of the pioneer teaching hospitals in Ethiopia, located in Amhara region, Northwest Ethiopia. The hospital has eight different laboratory sections, including Serology, which provides teaching, diagnostic, and research services for the university community, Gondar town inhabitants, and the nearby Woreda populations.

### 2.2. Study Participants

Following acquisition of informed consent, a total of 160 participants were included in this study. Out of them, 80 participants were diagnosed as positive for syphilis infection by the routinely used technique in the study area (ECOTEST-RPR) at the University of Gondar Teaching hospital Laboratory. Charts of all patients were reviewed to assess if specific therapy was commenced. Likewise, 80 apparently healthy individuals that do not have any history of sexually transmitted diseases were recruited from Gondar Blood Bank Center.

### 2.3. Specimen Collection and Processing

Blood sample was collected from each participant and centrifuged until the serum was separated, and sera were stored at −20°C until the actual laboratory tests were performed. Before running RPR, ELISA, and TPHA, the stored serum samples were thawed at 37°C in a water bath until the ice formed becomes completely dissolved. Thereafter, RPR, ELISA, and TPHA were done and results were achieved according to the kit's manufacturer's instructions. A specimen that shows an equivocal result for any of the tests was retested. This study was performed according to the Standards for Reporting Diagnostic Accuracy Studies (STARD) essential items for reporting diagnostic accuracy studies (http://www.equator-network.org/reporting-guidelines/stard).

#### 2.3.1. ECOTEST-RPR

[Principle] The antigen used in the ECOTEST-RPR (Assure Tech, Hangzhou, China) kit is a modification of VDRL antigen, which contains microparticulate charcoal to enhance the visual difference between a positive and negative result. Patient sera mixed with a fine particle cardiolipin antigen which has been enhanced with cholesterol, lecithin, and charcoal will result in a macroscopically visible flocculation-type precipitation if the patient's serum contains reagin—an antibody formed against cardiolipin (The detailed procedure can be accessed from the manufacturer's instruction, Assure Tech, Hangzhou, China.) Interpretation for each test was done using controls (positive and negative) according to the manufacturer's instruction as reactive (R), if clumping is seen, or nonreactive (NR)—smooth suspension, no clumping, or slight roughness.

#### 2.3.2. The DIALAB Syphilis IgG/IgM ELISA

[Principle] Using the antigen sandwich enzyme-linked method (ELISA), this syphilis IgG/IgM ELISA test (DIALAB, Germany) can detect anti-TP antibodies. Polystyrene microwell strips are precoated with recombinant *Treponema pallidum* antigens produced in *E. coli*. Recombinant TP antigens that are conjugated to horseradish peroxidase (HRP) conjugate are incubated in the microwells with the sample. The precoated antigens indicate the same epitopes as the HRP-conjugate antigens, but the hosts are different. If anti-TP is present in the sample during incubation, the conjugated and precoated antigens will be bound to the two-variable antibody domains, and what is captured on the solid phase is the specific antibody-antigen immunocomplex. It is important that chromogen solutions containing tetramethylbenzidine (TMB) and urea peroxide are added into the wells after the washing phase to remove sample and unbound conjugates. The colorless chromogens are hydrolyzed by the bound HRP conjugate to a blue-colored product when the antigen-antibody-antigen sandwich complex is present. At this point, the blue color turns yellow. This occurs after the reaction with sulfuric acid is stopped. What can be measured proportionally at this juncture is the amount of antibody in the sample with the amount of color. Colorless wells indicate negative anti-TP samples (The detailed procedure can be accessed from the manufacturer's instruction, DIALAB, Germany.)

(1) *Results Interpretation*. Each microplate should be considered separately when calculating and interpreting results of the assay, regardless of the number of plates concurrently processed. The results are calculated by relating each sample's optical density (OD) value to the cut-off value (CO) of the plate. If the cut-off reading is based on a single-filter plate reader, the results should be calculated by subtracting the blank well OD value from the print report values of samples and controls. In case the reading is based on a dual-filter plate reader, do not subtract the blank well OD from the print report values of samples and controls.

#### 2.3.3. Randox TPHA

[Principle] TPHA (Randox Laboratories, UK) reagents are used to detect human serum antibody to *T. pallidum* by means of an indirect hemagglutination (IHA) method. Preserved avian erythrocytes are coated with antigenic components of pathogenic *T. pallidum* (Nichols strain). These test cells agglutinate in the presence of specific antibodies to *T. pallidum* and show characteristic patterns in microtitration plates. Any nonspecific reactions occurring are detected using the control cells, which are avian erythrocytes, not coated with *T. pallidum* antigens. Nonspecific reactions may also be absorbed out using these control cells. Antibodies to nonpathogenic treponemes are absorbed by an extract of Reiter's treponemes, included in the cell suspension. Test results are obtained in 45–60 minutes, and the cell agglutination patterns are both easily read and are long-lasting. (The detailed procedure can be accessed from the manufacturer's instruction, Randox Laboratories, UK.)

(1) *Interpretation of Results*. When the test well is positive, the control well should be observed. The control cells should settle to a compact button. They should not be used as a comparison for nonreactive serum patterns since the control cells will give a more compact pattern than the test cells will. Agglutination in the control well indicates the presence of nonspecific agglutinins in the sample; the test should be reported as invalid. A serum that gives this result may be absorbed using the control cells as detailed under nonspecific absorption. A doubtful reaction with test cells should be reported as indeterminate.

### 2.4. Statistical Analysis

The data was cleaned and double entered on an Excel Spreadsheet and transported to SPSS version 20. JavaStat two-way contingency table analysis software (http://statpages.org/ctab2x2.html) was also used to calculate sensitivity, specificity, and predictive and kappa values. The test findings of ECOTEST-RPR and DIALAB-ELISA were compared with results of the reference method (Randox-TPHA). The kappa value was determined to evaluate the agreement between ECOTEST-RPR, DIALAB-ELISA, and Randox-TPHA.

## 3. Results

A total of 160 participants were involved in this study. 80 (50%) of them were diagnosed with syphilis using RPR as a diagnostic test in the study area. However, 80 (50%) of them were apparently healthy participants and were negative for syphilis by all types of tests (ECOTEST-RPR, DIALAB-ELISA, and Randox-TPHA). The participants' age range was from 20 to 52 years old, and most of them (77%) were between 22 to 32 years. Among the participants, 84 (52.5%) of them were males and 76 (47.5%) were females. Most of the study subjects (107, 66.9%) were from rural areas of the nearby inhabitants, and 53 (33.1%) of the subjects were urban residents (Table 1). Among 40 patients who were diagnosed as having syphilis by the RPR test, 2 patients had primary syphilis, and 9 patients had secondary syphilis with clinical presentation of nonitchy maculopapular rash, condyloma lata, and generalized lymphadenopathy whereas clinical data of the rest RPR-positive patients were not fully documented on the medical charts.

The sensitivity, specificity, and predictive values of ECOTEST-RPR and DIALAB-ELISA in this study were evaluated by using Randox-TPHA as a gold standard for the diagnosis of syphilis. Thus, the sensitivity and specificity of ECOTEST-RPR for syphilis detection were 100 and 80.8%, respectively. The positive predictive value (PPV) and negative predictive value (NPV) were 76.2 and 100%, respectively. The agreement between Randox-TPHA and ECOTEST-RPR tests was good with a kappa value of 0.88 (Table 2).

Moreover, the sensitivity and specificity of DIALAB-ELISA for syphilis detection were 98.4 and 94.9%, respectively. The positive predictive value (PPV) and negative predictive value (NPV) were 92.3 and 98.9%, respectively. The agreement between TPHA and ELISA tests was nearly perfect with a kappa value of 0.96 (Table 3).

In this study, we have revised the medical charts of each participant and found that all ECOTEST-RPR-positive patients were commenced appropriate medication. Moreover, we found two samples with equivocal test results for DIALAB-ELISA but reanalysis of these samples by DIALAB-ELISA and Randox-TPHA provides a positive result under both tests. Similarly, we reported 15 discrepant results (i.e., ECOTEST-RPR-positive but DIALAB-ELISA-negative) as a negative result following reanalysis and verified as negative by Randox-TPHA.

## 4. Discussion

Syphilis infection can be diagnosed using either the treponemal or the nontreponemal approach. Nucleic acid amplification techniques (NAAT) like polymerase chain reaction (PCR) have opened the way for the development of highly sensitive and specific point-of-care tests. Nevertheless, the use of NAAT methods in developing countries is limited due to their affordability and cost and the complexity of the techniques to be used by the existing human resources in diagnostic areas. Additionally, a definitive syphilitic diagnostic test based on the detection of *Treponema*-specific IgG has been made available. However, a nontreponemal technique like RPR is the most widely used including the study area, though it is unreliable as a positive result does not necessarily indicate treponemal infection. Thus, its application in screening blood donors, pregnant women, and social workers has been in question. Therefore, we assessed the performance of ECOTEST-RPR and DIALAB-ELISA assay with Randox-TPHA as a reference standard diagnostic test for syphilis.

In the current study, the overall sensitivity and specificity of ECOTEST-RPR as compared to Randox-TPHA were 100% and 80.8%, respectively. In our study, the sensitivity and specificity of RPR were comparable to the findings reported from Portugal, Korea, and Nepal [19–21]. However, sensitivity alone was much higher compared to reports from several studies [22–26]. The highest (100%) sensitivity in our finding could be as follows: First, our participants may not have only syphilis infection, suggesting cross-reactivity of ECOTEST-RPR with cholesterol, lecithin, and cardiolipin antigens found in other disease processes as a result of cellular destruction. Second, it may be due to variation in protocols from different companies as well; in fact, it should not be forgotten that the diagnostic performance of RPR is profoundly affected by the stages of syphilis infection, which is not fully addressed in our study. Based on our finding, the highest sensitivity (100%) is considered as a limitation of the ECOTEST-RPR serological test as its false positivity is enormous due to cross-reactive antibodies. Also, the chance of missing syphilitic cases at different stages is there because of the prozone effect. This high sensitivity of RPR has a negative implication in that wrongly diagnosed individuals are supposed to take medication together with their sexual partners (if there); this phenomenon results in economical, drug resistance, and social impacts in the community. Besides, blood from donors that are incorrectly diagnosed and labeled as positive will be discarded. Therefore, in the presence of this limitation and with the availability of other syphilis screening tests, the sole utilization of ECOTEST-RPR assay routinely as a diagnostic method remains of great concern in the study area.

Nevertheless, the specificity of the test in our study showed lowest performances compared to the Indian and South African report (96.96% and 100%, resp.) [22, 27] and incomparably very high with respect to findings from Turkey and Latvia (0%) [23, 28]. In contrast to findings from studies, those come up with a superior specificity of RPR [20, 27, 28], and we found a low specificity of manual ECOTEST-RPR test performance. The likely explanation may be the variation in the type of methods between our study and theirs. Although RPR is generally a nontreponemal test, we used a conventional manual ECOTEST-RPR test method while their method was the automated RPR test, indicating that the automated one has reduced interpersonal differences and other confounders. Furthermore, the clinical stage of study participants (primary, secondary, and tertiary syphilis) affects the specificity of the test because of the prozone phenomenon. Moreover, since reporting of the RPR test result (as positive or negative) is based on observation, the subjective (interpersonal) variation and subsequent decisions between laboratory analysts potentially affect the specificity of the test result.

Following the innovative introduction of ELISA and recombinant DNA (rDNA) technology for the diagnosis of syphilis, the traditional two-step approach of first screening with a nontreponemal test and then using a *Treponema*-specific confirmation test has been confronted and, as result, two diagnostic thoughts appeared. One school promotes the use of EIA as a screening test which needed to be confirmed by another test (technology or method but with equal or higher specificity), and the other school proposes the traditional approach of an algorithm [29].

Accepting either the traditional or the other approach, evaluating the performance of ELISA with reference to a better method is compulsory. In contrast to the ECOTEST-RPR test, DIALAB-ELISA has come up with a better test performance with reference to Randox-TPHA. In this study, we found 5 false positives with the DIALAB-ELISA test, thus giving a false-positive diagnostic rate of 8%; this appears to be the least value as compared to that of RPR (31%).

The sensitivity and specificity of DIALAB-ELISA with respect to Randox-TPHA are 98.4% and 94.9%, respectively. Findings obtained from several studies have come up with a sensitivity and specificity ranging from 90 to 100%, by far a result that is aligned with our study [23, 28–32]. Despite these similarities, it should not be forgotten that other findings [33–35] on the performance of ELISA moderately differ from the results of the current study. The most probable reason for this performance variation may be due to the type of immunodominant syphilis proteins that are incorporated into the wells of ELISA kits.

During the evaluation of comparative prediction of a diagnostic kit with a reference standard, several issues will influence the interpretation of their results of which the prevalence/magnitude of a disease is the most important factor [36].

In the current study, the positive and negative predictive values of ECOTEST-RPR are 76.2 and 100%, respectively. In the presence of variability on study participants (considering 50% of participants were positive for ECOTEST-RPR in this study), several studies come up with predictive values of ECOTEST-RPR similar with those of the current study [19, 21, 26] whereas different prediction values of ECOTEST-RPR have been reported from other studies [23]. The PPV and NPV of DIALAB-ELISA were 92.3% and 98.9%, respectively, in this study. Likewise, reports from studies showed PPVs and NPVs which are comparable with ours [23, 29, 34], whereas variable predictive values were seen from a Turkish study [23]. As we have stated earlier, the predictive values in this study (for ECOTEST-RPR and DIALAB-ELISA) were comparable and variable while comparing with PVs from several studies; a crucial point is that prevalence affects the PVs of any test. This implies that the same diagnostic test will have a different predictive accuracy according to the clinical setting/nature of study participants; thus, we strongly enlighten that the recommendations made in this study are based on the 50% magnitude of syphilis and that other studies should recognize the influence of the prevalence of disease when considering predictive values of diagnostic or screening tests.

The reported test performance of DIALAB-ELISA (sensitivity, specificity, and predictive values) in this study is encouraging and makes the test a better choice of the diagnosis approach in syphilis-suspected cases. Moreover, the agreement between DIALAB-ELISA and TPHA was nearly perfect (kappa value 0.96) as compared to that of ECOTEST-RPR and Randox-TPHA (kappa value of 0.88). Besides the superior performance, the ELISA technique has plenty of advantages over conventional flocculation screening tests (RPR). The method is designed potentially to be automated plus the reading of results is usually carried out by a microtiter plate reader, thus making the interpretation of results objective, unlike that of the RPR test which is subjective and hence requires having extensive experience. Unlike RPR, concerns like the prozone phenomenon and stage of syphilis infection do not affect the ELISA method. Regardless of all the above rational benefits, we want to enlighten the readers not to underestimate the drawbacks of ELISA methods.

## 5. Conclusion

This study comes up with a characteristically variable diagnostic performance of the DIALAB-ELISA test and the currently available traditional ECOTEST-RPR test in Ethiopia as compared to Randox-TPHA. ELISA kits with the recombinant *T. pallidum* antigens have certain attractions as a diagnostic tool. However, we cautioned the efficacy of the independent application of both ECOTEST-RPR and ELISA as a screening/diagnostic test for syphilis infection. In addition, it is important to underline that healthcare providers must perform a thorough review of each patient's clinical and treatment history while choosing the type of test and interpreting the results of RPR and ELISA IgG/IgM tests for syphilis diagnosis. Consequently, thorough studies should be conducted, aiming for a change of the current diagnostic scheme used in the community.

## Figures and Tables

**Table 1:** The detection of syphilis by Randox-TPHA reactivity among study participants at University of Gondar Hospital, 2015–2016.

Demography		Total (%)	Randox-TPHA Positive (%)	Randox-TPHA Negative (%)	COR (95% CI)	AOR (95% CI)
Gender	Male	84 (52.5)	33 (39.3)	51 (60.7)	1.11 (0.58–2.10)	1.15 (0.59–2.25)
	Female	76 (47.5)	28 (36.8)	48 (63.2)	1	1 1111111
Age (years)	20–30	121 (75.6)	48 (39.7)	73 (60.3)	0.76 (0.35–1.66)	0.75 (0.34–1.67)
	31–40	36 (22.5)	12 (33.3)	24 (66.7)	1	1
	≥41	3 (1.9)	1 (33.3)	2 (66.7)	N/A N/N/A	N/A
Residence	Urban	53 (33.1)	19 (35.8)	34 (64.2)	0.86 (0.44–1.71)	0.90 (0.44–1.83)
	Rural	107 (66.9)	42 (39.3)	65 (60.7	1	1

N/A: not considered during analysis; TPHA: *T. pallidum* hemagglutination assay; COR: crude odds ratio; AOR: adjusted odds ratio; CI: confidence interval; %: percent.

**Table 2:** Serology results of the ECOTEST-RPR and Randox-TPHA tests at the University of Gondar Hospital, 2015–2016.

ECOTEST-RPR	Randox-TPHA		
	Reactive	Nonreactive	Total
Reactive	61	19	80
Nonreactive	0	80	80
Total	61	99	160

Sensitivity of ECOTEST-RPR (61 of 61 samples), 100%; specificity of ECOTEST-RPR (80 of 99 samples), 80.8%; agreement (61 + 80 = 141, of 160 samples), 88%; positive predictive value (61/80) 76.2%; and negative predictive value (80/80) 100%.

**Table 3:** Serology results of the DIALAB-ELISA and Randox-TPHA tests at the University of Gondar Hospital, 2015–2016.

DIALAB-ELISA	Randox-TPHA		
	Reactive	Nonreactive	Total
Reactive	60	5	65
Nonreactive	1	94	95
Total	61	99	160

Sensitivity of DIALAB-ELISA (60 of 61 samples), 98.4%; specificity of DIALAB-ELISA (94 of 99 samples), 94.9%; agreement (60 + 94 = 154, of 160 samples), 96.25%; positive predictive value (60/65) 92.3%; and negative predictive value (94/95) 98.9%.

## Data Availability

The authors confirm that all data supporting our findings are contained within the manuscript and are fully available without restriction.

## Abbreviations

CIs: Confidence intervals
EIA: Enzyme immunoassay
ELISA: Enzyme-linked immunosorbent assay
FTA-abs: Fluorescent treponemal antibody absorption test
IgG: Immunoglobulin G
IRB: Institutional Review Board
NPV: Negative predictive value
NAAT: Nucleic acid amplification techniques
PCR: Polymerase chain reaction
PPV: Positive predictive value
RPR: Rapid plasma reagin
rDNA: Recombinant DNA
STARD: Standards for Reporting Diagnostic Accuracy Studies
STD: Sexually transmitted disease
TPHA: *T. pallidum* hemagglutination
*T pallidum*: *Treponema pallidum*
UGH: University of Gondar Hospital
VDRL: Venereal disease research laboratory.
